# Clinico-anesthetic changes following administration of propofol alone and in combination of meperidine and pentazocine lactate in dogs

**DOI:** 10.14202/vetworld.2016.1178-1183

**Published:** 2016-11-02

**Authors:** A. K. Anandmay, L. L. Dass, A. K. Sharma, M. K. Gupta, K. K. Singh, B. K. Roy

**Affiliations:** 1 Touring Veterinary Officer, Department of Animal Husbandry, Adardih Block, Saraikella - 832 401, Jharkhand, India; 2 Department of Veterinary Surgery and Radiology, Ranchi Veterinary College, Birsa Agricultural University, Ranchi - 834 006, Jharkhand, India; 3 Department of Veterinary Pathology, Ranchi Veterinary College, Birsa Agricultural University, Ranchi - 834 006, Jharkhand, India; 4 Department of Veterinary Pharmacology and Toxicology, Ranchi Veterinary College, Birsa Agricultural University, Ranchi - 834 006, Jharkhand, India

**Keywords:** clinico-anesthetic changes, dog, meperidine, pentazocine, propofol

## Abstract

**Aim:**

The aim of this study is to find out the effect of propofol and its combination with meperidine and pentazocine lactate on certain clinico-anesthetic profiles in dogs.

**Materials and Methods:**

15 apparently healthy mongrel dogs of either sex of about 1 year of age were randomly divided into three groups of five dogs each. The animals of Group I were administered propofol intravenously alone “to effect,” whereas meperidine at 2 mg/kgb.wt. and pentazocine lactate at 2 mg/kg b.wt. were injected intramuscularly 15 min before propofol “to effect” in Groups II and III, respectively. Atropine sulfate at 0.04 mg/kgb.wt. was injected intramuscularly 20 min before each treatment. Rectal temperature, heart rate, respiration rate, and anesthetic indices were recorded before and at 5, 10, 20, 30, and 60 min of induction.

**Results:**

As compared to Group I, the animals of Groups II and III exhibited a significant decrease (p<0.05) in the level of rectal temperature, respiration rate, and heart rate. Duration of recumbency, time of standing, time of recovery as well as the duration of analgesia were longer in pentazocine lactate (Group III) followed by meperidine (Group II) as compared to propofol alone (Group I). Meperidine treated dogs showed defecation and muscle twitching during anesthesia.

**Conclusion:**

Meperidine and pentazocine are suitable opioids used in combination with propofol for achieving surgical anesthesia and helpful in reduction of propofol dose.

## Introduction

Propofol, an alkyl phenol (2, 6 di-isopropylphenol), has been developed as oil in water emulsion because the compound has limited water solubility. Total intravenous anesthesia (TIVA) protocols with propofol are widely used in medical neuroanesthesia [1]. Propofol produces anesthesia characterized by rapid onset, short duration, lack of accumulation on repeated administration, and lack of excitatory effects on induction, during maintenance and recovery. Recoveries in dogs are reported to be smooth in propofol anesthesia [2]. Respiratory depression and apnea are the most common adverse effects associated with IV administration of propofol [3] along with pain on injection [4].

Meperidine is a synthetic opiate agonist (Kappa opioid receptor) belonging to phenyl piperidine class. The onset of analgesic action of meperidine is slightly more rapid than morphine and duration of action is slightly shorter. Meperidine is more respiratory depressant as compared to morphine.

Pentazocine is a benzomorphan derivative which has both agonistic action (Kappa opioid receptor) and weak antagonistic or, partial agonistic action (µ opioid receptor) [5]. Side effects of pentazocine are respiratory depression, dizziness, nausea, vomition less common than morphine and increase in heart rate along with blood pressure when given in high doses [6]. Propofol has been used in combination with remifentanil [7], fentanyl, and butorphanol [8] in human practice and as admixture with alfentanil in dogs [9]. Combination of IV anesthetic with opioid analgesics have been used for achieving balanced anesthesia with reduced side effects and promote earlier recovery time and less post-operative nausea and vomiting [10]. Opioids are combined with other anesthetic to produce ideal anesthetic protocol [11,12].

There is paucity of data regarding the use of pentazocine and meperidine as preanesthetic to propofol in dogs. Hence, the present paper deals the clinico-anesthetic changes following administration of opioids as preanesthetic in propofol anesthesia in atropinized dog.

## Materials and Methods

### Ethical approval

This research project has been approved by the Ethical Committee.

### Animals

15 apparently healthy mongrel dogs of either sex of about 1 year of age were used in this prospective clinical trial and divided randomly into three groups of five dogs each based on the anesthetic regimen given. The animals were dewormed, vaccinated, and maintained in an isomanagemental condition. They were kept off feed and water for 12 and 6 h, respectively, before the start of the treatment.

### Anesthetic protocols

In all the three groups, atropine sulfate (at 0.04 mg/kgb.wt., IM) was administered 20 min before each treatment. Anesthesia in Group I was achieved by propofol alone (IV “to effect”), whereas, in Group II, it was produced by meperidine (at 2 mg/kgb.wt., IM) and propofol (IV “to effect”) sequentially at 15 min interval. Anesthetic protocol used in Group III was similar to that of Group II, but instead of meperidine, pentazocine was administered intramuscularly at the dose rate of 2 mg/kg b.wt. Propofol “to effect” was injected in each animal of the three groups to produce general anesthesia till the loss of pedal reflex which served as a guide for the development of surgical anesthesia.

### Evaluation of clinico-physiological parameters

The baseline values (0 h) for clinical and physiological parameters were recorded before injection of the drug in each animal of each group. Physiological parameters such as rectal temperature (°F), respiratory frequency (breaths/min), and heart rate (beats/min) were recorded at 0 min and then at 5, 15, 30, and 60 min following induction of anesthesia. Various reflexes such as corneal, palpebral, pedal, anal, and cutaneous were also recorded during the observation period.

### Evaluation of anesthetic indices

The anesthetic indices such as onset of analgesia (time interval between initial bolus injection of propofol to disappearance of pedal reflex), duration of analgesia (time interval between the loss and return of pedal reflex), duration of recumbency (time intervals after initial bolus injection of propofol to the dog’s assumption of sternal posture), time to standing (time interval between assumption of sternal posture and dog’s ability to stand), and recovery time (period between the last bolus injection or cessation of infusion of propofol and dogs ability to stand as judged on the basis of physical symptoms and reflexes). Anesthetic indices were recorded in minutes except onset of anethesia and durationof recumbency, which were calculated in seconds. Total dose of propofol for induction (mg/kg) was also calculated in each group.

### Statistical analysis

One-way analysis of variance and Duncan multiple range test were used to compare the means at different intervals among the groups as per method described by Snedecor and Cochran [13]. The level of significance was set at p<0.05.

## Results

### Clinico-physiological parameters

There was asignificant decrease in rectal temperature (p<0.05) in Groups II and III at 5 and 10 min postinduction which progressively increased and reached to the base value at 60 min of observation (Table-1). Contrarily, a non-significant decrease in rectal temperature could be recorded in Group I (Control) throughout the period of observation. The maximum fall in rectal temperature was observed at 10 min in Groups Iand III and at 5 min in Group II as compared to their base value. Rectal temperature of Group II differed significantly to that of Group I at 5 min, whereas Groups III and I differed significantly between themselves at 30 min postinduction. In all the groups, the rectal temperature reached to preinjection value at 60 min of observation.

**Table 1:** Mean±SE value of rectal temperature (°F) of different groups at different time intervals of observation.

Group	Period of observation (in min)					

	0	5	10	20	30	60
I	100.87±0.13	100.56±0.23^A^	100.53±0.47	100.60±0.47	100.68±0.41^A^	101.10±0.34
II	101.40±0.22^a^	99.58±0.26^Bb^	99.72±0.10^b^	100.80±0.14^ab^	100.94±0.14^ABa^	101.00±0.24^a^
III	101.25±0.17^a^	100.30±0.1A^Bb^	100.20±0.18^b^	101.16±0.20^a^	101.61±0.33^Ba^	101.37±0.42^a^

Values with same superscripts in a column (capital letters) did not differ significantly (p>0.05). Values with same superscripts in a row (small letters) did not differ significantly (p>0.05). SE=Standard error

Significant fall (p<0.05) in respiration rate was observed at 10 min postinduction in Groups I and II and at 20 min in Group III postinduction and reached near to the baseline value at 60 min of observation (Table-2). In contrast to this, Group I showed a nonsignificant decrease during peak of anesthesia. Group-wise analysis of data revealed that Group I differed significantly with that of Groups II and III at 5 and 10 min postinduction, respectively. However, Groups I and II also varied significantly between themselves at 10 min of observation.

**Table 2:** Mean±SE value of respiration rate (per min) of different groups at different time intervals of observation.

Group	Period of observation (in min)					

	0	5	10	20	30	60
I	23.80±0.72	22.40±0.61^A^	21.80±0.77^A^	22.20±0.72^A^	23.40±0.61	23.60±0.61
II	23.20±1.04^a^	19.60±0.88^Bbc^	18.40±1.15^Bb^	20.80±1.25^Aabc^	23.00±0.94^ac^	23.40±1.15^a^
III	21.56±1.88^a^	21.45±0.44^ABa^	19.00±0.44^Bab^	17.22±1.00^Bb^	21.80±0.80^a^	22.02±0.28^a^

Values with same superscripts in a column (capital letters) did not differ significantly (p>0.05). Values with same superscripts in a row (small letters) did not differ significantly (p>0.05). SE=Standard error

A significant decrease in heart rate (p<0.05) was recorded in Group I at 5 and 10 min postinduction followed by a progressive increase in heart rate tending to reach near the base value could be recorded at 60 min of observation (Table-3). In contrast to Group I, Group II animals showed a nonsignificant increase throughout the observation period. After an initial decrease up to 10 min, Group III animals showed an increasing trend up to 60 min of observation. The maximum decrease in heart rate in Groups I and III was observed at 10 min postinduction. Group-wise analysis of data revealed a significant variation in heart rate at 5 min of observation among Groups I, II, and III, whereas Groups I and II differed significantly at 10 and 30 min of observation.

**Table 3:** Mean±SE value of heart rate (beats/min) of different groups at different time intervals of observation.

Group	Period of observation (in min)					

	0	5	10	20	30	60
I	106.00±3.11^a^	90.20±4.33^Ab^	88.40±3.34^Ab^	100.00±2.26^ab^	102.60±4.45^Aab^	100.40±7.99^ab^
II	105.80±5.12	116.60±2.07^B^	117.60±3.12^B^	115.20±4.74	114.80±5.62^B^	106.20±4.62
III	100.20±2.97	98.00±2.59^C^	95.00±2.93^AB^	100.60±3.53	101.20±2.73^A^	101.80±1.84

Values with same superscripts in a column (capital letters) did not differ significantly (p>0.05). Values with same superscripts in a row (small letters) did not differ significantly (p>0.05). SE=Standard error

Pedal reflex disappeared at 36.00±1.86, 33.00±2.56, and 30.00±2.74 s after induction of anesthesia and reappeared after 10.26±1.21, 11.80±0.92, and 12.38±0.87 min in Groups I, II and III, respectively. All the reflexes were present before induction with propofol in all the groups. Pedal reflex followed by corneal, palpebral, cutaneous, and anal reflex disappeared in all the groups following induction of anesthesia with propofol. Corneal, palpebral, and cutaneous reflex reappeared within 30 min postinduction in all the groups. Anal reflex appeared within 20 min in Groups I, II, and III.

### Anesthetic indices and induction dose of propofol

The mean time of onset of analgesia following administration of propofol was recorded maximum in Group I (Table-4). Quick onset of analgesia was observed in Group III followed by Group II as compared to Group I. The longest duration of analgesia was observed in Group III followed by Groups II and I.

**Table 4:** Mean±SE value of anesthetic indices and induction dose of propofol of different groups.

Group	Onset of analgesia(s)	Duration of recumbency(s)	Duration of analgesia (min)	Time of standing (min)	Time ofrecovery (min)	Induction dose of propofol (mg/kg)
I	36.00±1.86^a^	32.60±1.50^a^	10.26±1.11^a^	13.60±1.21^a^	15.93±1.17^a^	5.93±0.06^a^
II	33.00±2.56^ab^	31.20±1.16^a^	11.80±0.92^ab^	15.55±1.48^ab^	18.40±1.21^a^	5.43±0.16^a^
III	30.00±2.74^ab^	28.90±1.96^a^	12.38±0.87^ab^	15.40±0.51^ab^	17.20±0.86^a^	5.27±0.10^b^

Values with same superscripts in a column (small letters) did not differ significantly (p>0.05). SE=Standard error

The maximum decrease in duration of recumbency was observed in Group III followed by Group II as compared to Group I. Groups I, II, and III did not differ significantly among themselves (Table-4). The mean time of standing in Groups II and III showed a nonsignificant increase as compared to Group I. The maximum increase in time of standing was observed in Group II followed by Groups III and I. Time of recovery could be observed maximum in Group II followed by Groups III and I.

### Induction dose of propofol

Induction dose of propofol increased significantly (p<0.05) in Groups I and II as compared to Group III (Table-4).

## Discussion

Propofol provides dependable short anesthesia in combination with opioids or tranquilizer or alpha2agonist for surgical procedures such as castration, ear flushing, ultrasound examination, biopsies, and suturing of small lacerations. Propofol is sedative/hypnotic and has only minimum analgesic action at a subanesthetic dose. As with other hypnotics, even when an animal is rendered unconscious with propofol, it will respond to painful stimuli unless analgesic drugs such as morphine or medetomidineare used in combination with propofol [14]. Pain caused due to IV administration of propofol is also minimized by administration of opioids [14]. Propofol as sole agent was generally unsatisfactory because of its poor analgesic property [15]. Meperidine block the sodium channels and inhibit activity in dorsal horn neuron in a manner analogous to local anesthetics [16]. Meperidine also exerts effecton alpha2 receptor suggesting that it may possess some alpha2agonist-like properties [17]. Pentazocine is classified as agonist-antagonist opioids and is clinically similar to butorphanol. They induce mild analgesia accompanied by minimal sedation, respiratory depression, or adverse cardiovascular effects. Following induction of anesthesia, the clinical observation revealed hypothermia in both the groups. Murrell *et al*. [18] and Anandmay *et al*. [19] also observed similar finding during propofol recovery in canines. The fall in rectal temperature may be a sequel to thermoregulatory depression and decreased metabolic rate due to propofol which was further potentiated by meperidine and pentazocine.

Propofol can induce significant depression of respiratory function characterized by a reduction in the rate of respiration. Opioids in combination with propofol increase the probability of respiratory depression during anesthesia [20]. Keates and Whittem [21] observed apnea after induction of anesthesia with propofol and depends on the rate of administration. Contrary to this, apnea was absent in this study which reveals that the dose as well as the rate of administration of propofol was sufficient to produce satisfactory induction of anesthesia without apnea.

Opioid administration causes respiratory depression and apnea [15]. Ahlgreneand Stephen [22] along with Pandey and Sharma [23] observed respiratory depression by pentazocine in dog, which supports the finding of this study. Pentazocine is a synthetic narcotic that design to have lower rate of respiration [24]. Dahan *et al*. [25] stated that buprenorphine has also the property of respiratory depression and is due to its partial µ agonistic activity in human. Buprenorphine being a partial µ opioid agonist may have a wider safety profile compared to full µ agonist, especially with regards to respiratory depression. A nonsignificant change in respiration rate in group I was found. Similar result was also observed by various workers in canine [12,26,27]. In contrast to this, a significant decrease in respiration rate was observed by Lerche *et al*. [28] in dogs and Khameneh *et al*. [29] in rabbit with propofol. The respiratory depression may be due to the respiratory depressant activity of propofol which was further potentiated by meperidine and pentazocine, in accordance with the findings of Covey-Crump and Murison [30].

Cardiovascular changes induced by propofol administration consist of a slight decrease in arterial blood pressures (systolic, diastolic, and mean) without a compensatory increase in heart rate [20]. Hypotension is primarily the result of arterial and venous vasodilation. Propofol does not depress baroreflex sensitivity directly but may produce an increased vagal tone and decrease in sympathetic tone by central mechanism [31]. Rate of administration of propofol may influence the cardiovascular system potentially resulting in hypotension [21]. In this study, slight increase in heart rate in Group III might be due to fact that meperidine hydrochloride has an atropine-like action [32].

The onset of action of anesthesia (induction time) was 36.00±1.86, 33.00±2.56, and 30.00±2.74 s in Groups I, II and III, respectively. It was interesting to note that the groups which received premedicants had a decreased mean induction time as compared to propofol alone. Decrease in induction time in Groups II and III might be due to synergistic effect of meperidine and pentazocine, respectively, in propofol anesthesia.

The most characteristic feature of propofol anesthesia was its quality of induction of anesthesia. Induction was smooth and quick in all the groups. The finding of the present study is in accordance with the reports of some workers [33,34].

Administration of premedicants had no any effect on quality of induction and almost all the animals of all groups recorded smooth, quick, and excitement free induction of anesthesia. These features of induction might be due to propofol’s inherent quality of induction [35]. In spite of milk-like appearance of propofol emulsion, it was free flowing and injected easily. Comparing the combination of different premedicants with propofol, meperidine appeared to be more effective followed by pentazocine in relation to onset of analgesia.

The reason for shorter duration of action in Group I was probably due to extrahepatic mechanism that contributed to rapid clearance of propofol from blood [33,34]. Recovery period was achieved by standing up and ambulation of the animals. Propofol’s high lipid solubility results in rapid and extensive redistribution, which contribute to termination of drug’s anesthetic effect [36]. The average recovery time for colonoscopy in human was shorter in patient receiving propofol alone as compared to propofol plus narcotics [37].

A significant decrease (p<0.05) in induction dose of propofol was recorded in Group III in comparison to Groups I and II may be explained by the fact that lowest dose of propofol required to induce surgical plane of anesthesia was in pentazocine followed by meperidine compared to unpremedicated dogs. Propofol administration is preceded by a preanesthetic such as morphine, fentanil, and remifentanil (opioids); the induction dose of propofol can be decreased substantially with cardiovascular stability [14,30,38,39]. The dose of induction of anesthesia in nonpremedicated dogs and cats ranges from 6 to 8 mg/kgbwtIV, whereas the dose in sedated animals may be as low as 2 mg/kgIV [40]. Amarpal *et al*. [41] found the induction dose of propofol to be 5.65±0.39 mg/kg which was reduced markedly by the use of premedicants such as xylazine and medetomidine. Combined use of opioids may reduce the respective doses of the drugs, as well as adverse reactions induced by their single use [42]. The decrease in induction dose of propofol might be due to premedication with pentazocine and meperidine.

## Conclusion

It is concluded that the meperidine and pentazocine lactate are the suitable opioids used as an analgesic in combination with propofol for achieving surgical anesthesia with minimal cardiorespiratory effects and helpful in reduction of propofol dose.

## Authors’ Contributions

AKA was responsible to conduct the work under M.V.Sc degree programme; LLD and AKS have designed the work and prepared the manuscript; MKG, KKS, and BKR involved collection of samples and critical revision of manuscript.
